# Foreign

**Published:** 1841-01-30

**Authors:** 


### FOREIGN.

Such general interest has of late been taken in the various reports furnished of the cure of strabismus by a section of the recti muscles of the eye, that we have felt it our duty thus far to lay them before our readers. The deformity is every where to be met with, and the operation for its relief is so simple, and so devoid of danger, that many practitioners are induced to undertake it, who would abstain from operations of greater magnitude. It is, however, of too recent a date to enable us to jndge yet of its curative powers: regular statistics, taken after a sufficient period has elapsed to confirm the permanency of the first results, can alone settle this point. We shall, therefore, probably defer further notice of the subject, until this test of time has been obtained; but, in order to render it complete up to the present period, we republish to day an article by Mr. Guthrie, which presents the most methodical condensation of the history of the operation, and the various modes of performing it which we have yet seen.

*The half yearly report laid before the Governors of the Royal Westminster Ophthalmic Hospital, on the 25th July*, 1840. By Charles W. G. Guthrie, Jun.—We are greatly pleased to see Mr. Charles Guthrie appear upon the scene. He is a young surgeon of much promise, and bids fair to succeed to the honourable position so justly held by his able father. We wish him the success which we cannot doubt he will obtain.

Mr. Guthrie observes:—

"The operation was proposed by Dr. Stromeyer of Hanover, but was first practised by Dr. Dieffenbach of Berlin, in January last: and Mr. Pyper, a student, who had been recommended to the notice of the learned Professor by my father, and had assisted Dr. Dieffenbach in several of his operations, caused the necessary instruments to be made for him on his return to London in March, and pointed out the different steps of the operation as he had seen them performed. The first operation was done on the 18th of April, but the instruments have since been considerably modified, and the operation has been rendered more simple, so that it may be readily accomplished in less than one minute, with the greatest precision and safety on an adult, offering no resistance; I have done it in a great many instances in half a minute, and it has even been done by Mr. Guthrie in a few seconds by one introduction of the small curved knife. But this method is not safe, for the ball of the eye may be injured by any irregular motion of the patient, which did occur in one unruly boy, but neither in this nor in any other instance has any evil result, or more than slight inflammation, followed the operation in the eighty-four cases in which it has been done in this hospital or in private life, nor in others in which I have assisted; and in no case has it failed in overcoming the immediate evil."

The degree of power, says Mr. G. which the person possesses over the eyes is occasionally very doubtful on the first inspection of them; both eyes squinting in turn, or together, and it is only after a little delay that the really defective eye can be clearly ascertained; whilst in others the motions of the eye are altogether so irregular, and so little under control, that it becomes doubtful which muscle should be divided, or whether it would be proper to attempt any operation at all. In most instances, persons have seen double for two or three days, or even for a longer time after the operation, but this gradually subsided in every instance, save one, as the axis of the affected eye began to correspond with that of the other. In the exception a slight cast can be perceived on a careful examination, so slight, however, as scarcely to be remarked. In one case the sound eye turns in more than the eye which has been operated upon, but vision is single. In four cases, the eyes seemed disposed to turn inwards again for a few days, but from this they eventually recovered. In one case it has been necessary to repeat the operation—in two a new pupil has been made—in almost all, vision has been in a greater or less degree gradually improved, although the amendment of the appearance has been in some the essential point gained. In a few cases, the sight of each eye appears to be equally good. In one case the eye turned, and continues turned a little outwards after the operation.

*Operation on Children.*—When a child under eight or ten years of age, or an unruly one who may be older, of which we have had one instance at seventeen, is the subject for operation, it must be secured in the same way as in the operation for cataract, by being laid on a narrow table and covered by a strong sheet, which ought to be tied firmly underneath. The head is to be placed on a pillow, and steadily held by a spare assistant, whilst another, if necessary, further secures the body; for children of four or five, are sometimes so strong, and resist so much after the operation has been begun, as to render it very difficult to complete it, unless perfectly mastered, when they become comparatively quiet. In the first case of this kind, the muscle was not divided, and the operation had to be repeated. The youngest child Mr. G. has operated on was two years and three months old, the two next youngest were four years old.

*Operation on Adults.*—" When a patient is an adult he may be placed in a high-backed chair with the head reclined. An assistant raises the upper lid in the gentlest manner, with the ordinary silver elevator used for the same purpose in cases of soft cataract in children, holding it with one hand nearly perpendicularly to the forehead, and desiring the patient to turn the eye *outwards* in a case of squinting *inwards*, fixes a strong double hook with the other hand into the tunica albuginea, through the conjunctival membrane a little distance from the cornea, in the middle line of the eye, or what is pedantically called its equator. The points of the hook are short that they may not enter, or scarcely enter, the sclerotic coat, but they are long enough to pierce the tunica albuginea, for if they only penetrate the conjunctival membrane, this slips, and they raise it from the ball and cause its elevation near the cornea, which delays the cure. The assistant must also take care that the elevator duly raises the lid and gently confines it against the edge of the orbit in such manner that it may not slip, nor allow the upper conjunctive fold or part of the internal portion of the lid to bulge out below it, which will be apt to occur unless it is carefully prevented, by holding the elevator in the manner directed. On the right eye the elevator is to be held in the left hand, the hook with the right, and vice versa. The operator (or an assistant) having depressed the under eyelid with the fore or second finger of the left hand, directs the assistant to draw the eyeball gently outwards with the hook, until the semilunar fold of the conjunctival membrane begins to yield to the traction, when it should be held perfectly steady on the middle line, the centre of the pupil being directly under the shaft of the hook. He then makes an incision nearly equidistant from the hook, and the edge of the semilunar fold, through the conjunctival and the cellular membrane which may intervene between it and the tendon of the rectus muscle, directly upwards and downwards, and inwards towards the orbit. Some surgeons use a small straight knife for this purpose, some a pair of scissors, in which case the conjunctival membrane may be raised by a pair of eye forceps at the semilunar valve, and the fold thus formed between it and the hook divided, but I generally cut through this part at once with a small curved knife, which is introduced under the conjunctiva, from below the line of the hook, the point being brought out upwards through the membrane, or with scissors convex on the lower edge, and cutting sharply up to the points which are blunt. The incision is to be enlarged if necessary to at least three-eighths of an inch in length, which exposes the tendon of the muscle going to be implanted into the outer or sclerotic coat, and which is made more distinct by the point of the knife or scissors whichever are used, or by the blunt end of the small, flat, curved and slightly grooved director, for which the knife is to be exchanged, and any blood which flows is to be taken up with a small piece of sponge. If the incision is made too close to the semilunar valve, the subjacent cellular membrane frequently becomes infiltrated with blood, and prevents the muscle from being seen, which does not occur when the incision is made at a greater distance from it, but this need not delay the operation. The curved director is now to be introduced by a gentle steady motion beneath the tendon, carrying it inwards rather deeply through the cellular and fatty membranes, so as to be passed under the muscular, as well as the tendinous part, which causes the eye to roll a little inwards: and which should not be prevented by holding the hook too firmly. The point is then to be raised by depressing the handle, when it will appear at the upper part of the incision, having the muscle on its grooved surface; and this elevation of the tendinous attachment of the muscle turns the eyeball outwards, so that the hook is, in fact, no longer necessary, and may be dispensed with after the first incision is made through the conjunctival membrane: and in very determined persons it may be dispensed with altogether. The director being now held in the left hand over the lower eyelid, the curved knife is to be run along the slightly marked groove of the director, and the muscle becoming tendinous is divided. The operation may be done throughout with the same pair of blunt ended curved scissors with which it was begun, the lower convex limb being gradually introduced under the muscle in the same manner and with the same precautions as the director. When the muscle is fully divided there is usually a small spurt of blood, which I consider a satisfactory sign, but which soon ceases to flow, and the eye is generally observed to turn a little outwards, but not always forcibly. The elevator should then be removed, when the upper eyelid falls, and the eye is to be sponged clean. The patient should now be desired to open the eye by raising the lid, and to turn the eye inwards; if he cannot do this the operation has been completed, but if he can do it in the slightest degree, the muscle or its lateral cellular or tendinous attachments have not been entirely divided, and the eye being again secured by the fore-finger or the elevator, the scissors are to be used, or the director is to be once more introduced, and the undivided part sought for and incised, when it will be found that the patient can no longer turn the eye inwards, and it is surprising how small a portion of attachment can do this, either on the under or upper part. In the earlier operations performed I was not aware of this circumstance, and in two cases in which a slight turn *inwards* re-appeared a fortnight after the operation, I attribute it to this portion of membrane, which in all probability adhered to the posterior end of the divided muscle. It may also effect this slight turn from its connexion either with the rectus superior above, or the inferior below, as the case may be. In three of the last cures performed the eyes would not turn outwards, although the sclerotic coat was rendered distinct to all present, for nearly three-eighths of an inch every way, which indeed ought always to be done to ensure success, and it was only by dividing the additional band of membrane or tendinous expansion I have alluded to in one case above, and in two below, that the operation was perfectly completed. The posterior cut end of the muscle is to be pushed backward by the director away from the other portion, and from the ball of the eye, so that it may unite indirectly to the posterior part of the globe and not to its side, and the edges of the incision in the conjunctival membrane are to be adjusted by the end of the same instrument. The eyes are then to be closed, by a small pad and a bandage, passed around the head; the pad of the eye operated upon should be kept wet with cold water for the first twenty-four hours, and the eye closed for two or three days. These precautions are not absolutely necessary, but it is as well to observe them, and to apply a few leeches to the lower lid, if there should be any pain and swelling. The cut edges of the conjunctiva are prone to retract and swell and become elevated, whilst a small projection or growth takes place from the sub-conjunctival cellular membrane, which requires to be touched from time to time by the sulphate of copper, or the nitrate of silver, or even to be cut off with the curved scissors. The eye is bloodshot occasionally for some days. The operation is but a trivial one, and is not apparently very painful, although some persons complain of it much more than others, as is usual with all operations on the eye."

Such is the operation as it is now very generally performed.

Mr. Liston uses scissors, and holds the lower lid down with a spring tenaculum. This operation is rather longer, and certainly rougher than the common one.

There have been several proposals for simplifying the operation. Most of them refer to the specula. We shall notice one or two.

Mr. H. Attenburrow, of Nottingham, a very dexterous young surgeon, says.*

"The short-curved instrument I use for the upper eyelid, gently raising the tarsus with the fingers, and passing it underneath, so that the lid is completely supported, and may be raised at any time, if required, from the ball of the eye. The handle of the speculum lies on the patient's forehead. The longer instrument is applied in the same way to the inferior tarsus, and, from having its handle of a proper length (about seven or eight inches,) the assistant's hand does not in the least interfere with the operator. With these specula I have been able to operate with ease on the most unruly children; and another advantage is, that the patient can be allowed to rest by removing the instrument in any stage of the operation. In making the specula, care should be taken to obtain a proper curvature of the hooked portion which is intended to hold the eyelid, as, on its malformation, great pain will result on its application; but any surgeon making the instrument had better apply it to his own eye, which will teach him the proper curvature. They are easily manufactured from copper wire, and the ends should be lacquered, which is effected by a spirit lamp."

In a note it is stated that—Coxeter of Grafton-street, has just invented a very simple and well-contrived apparatus for keeping down the lower lid without the aid of an assistant. It consists of a small speculum, by which the lower lid is held firmly, but gently downwards, and is retained in that position by means of a curved spring, fitted with a pad, which embraces the lower jaw.

Mr. Clay, of Manchester, has also invented a speculum. In general form and size it represents a teaspoon, having about one third of the extremity of the bowl removed by a semilunar cut; on the external surface it is less convex than a spoon; on the internal surface, that which is intended to be applied to the eye, it is concave and hollowed so as to fit the cornea and front of the globe, while the edges are thick to keep apart the eyelids. When placed in its position between the lids the inner angle of the eye is left uncovered, in consequence of the semilunar notch, and the surgeon is supposed to operate in the resulting space.

Mr. Adams proposes a knife of this sort:—it possesses all the properties of a scalpel, namely, utility, effectiveness, and safety, in a smaller space than any other instrument with which I am acquainted that has been adopted for dividing the internal rectus muscle: the blade

*Lancet, Sept. 12, 1840.

consists of two parts, a rounded portion, resembling the blunt part of a hernia knife, and a small flat sharp extremity, having a transverse cutting edge, which at one end terminates in a shoulder by uniting with a short sharp front edge, while its other extremity forms a point by meeting the back edge at right angles, so that we have the transverse effective part of the blade in immediate connection with the useful shoulder and point. The instrument described cannot be thrust into the eye by any sudden accidental resistance of the patient; and this safety is owing to the position of its straight cutting edge, which does not require any sharp part of the knife to be directed towards the globe of the eye during any moment of the operation: the muscle being slightly raised across the blunt hook, its fibres are divided transversely by the knife, its edges being directed towards the hook, but at right angles to it, so that the flat side of the blade is held towards the eye.*

But, after all, if we are to trust Mr. D. O. Edwards, these instruments are "all my eye." He, following the suggestions of Mr. French, does without them.

"The way in which I proceeded in all these was the following:—The patient was seated on a chair in a moderate light; an assistant standing behind covered the sound eye with one hand, and, with the other raised up the superior lid of the affected organ. The patient having been directed to turn the eye upwards and outwards, I snipped the conjunctiva at the under edge of the rectus, and passed a curved probe beneath the muscle: by depressing the handle of the probe the point was thrust forward, and appeared above the upper edge of the divided muscle, and by a cut of the scissors was enabled to emerge. The fibres were finally divided upon the probe.

The operation, thus abbreviated, consists of but three steps:—

First, the snipping of the conjunctiva.

Second, the introduction of the probe under the muscle.

Third, the cutting out of the probe.

The usual formidable notes of preparation are avoided. The probe which I employ consists of two stems placed parallel, at a distance sufficient for the point of the scissors to pass between. It is curved into a hook in the shape of a semicircle, of which the diameter is six lines. The pattern may be had at Savigny's or Fergusson's."†

Mr. Duffin, however, who informs us that he has operated *one hundred and seventy times*, and therefore ought to know something about it, laments the frequent failures which have happened, and thinks it not impossible that the operation may fall into disrepute. "When," says he, "it is imperfectly performed, a few fibres of the tendon, perhaps, or some apparently insignificant band of fibrous adhesion having escaped the scissors, the patient loses the power of turning the eye horizontally inwards, so as to bury the cornea in the nasal canthus to the same extent that he could do previously, and thus the inexperienced may be misled. If, in like manner, in his effort to accomplish this movement, it be found that the patient is still capable of directing the pupil either upwards or downwards, and only slightly inwards, we may rely upon it that the operation is incomplete, and that when the eye is left at rest, this modification of the original evil, will, in a minor degree, be found to persist. Now, the operator being satisfied, from the extent to which he may have laid bare the sclerotica, that he must have divided the whole of the tendon of the adductor, often erroneously concludes that this slight obliquity arises from sympathy, and that, in the course of time, it will entirely disappear. But, unless we can imagine that the unsevered parts will gradually relax, or become elongated, it is impossible that the eyeball can ever emancipate itself thoroughly from its confined position. I have lately examined several cases that were operated upon, When this mode of relieving strabismus was first practised in this country, and do not find that time has thus far effected any amelioration, nor do I think it at all probable that further improvement will ever take place; but the contrary.

When the operation is complete in every respect, the patient is wholly incapable of directing the pupil of the eye beyond the centre of the orbit, either in a horizontal or oblique line."*

When the eye can turn upwards and inwards, or downwards and inwards, it is probably because some tendinous or cellular attachments have been left undivided. They ought, therefore, to be perfectly severed.—*Medico-Chirurgical Review.*

---

*Ioduret of Iron in the treatment of Syphilitic Ulcers.*—M. Baumes, head surgeon of the hospital at Lyons, has of late used this new ferruginous preparation with most satisfactory results in the treatment of old and obstinate syphilitic ulcers, especially when the state of the system of the patient was at the same time feeble and scrofulous. He administered it in the form of pills with Thebaic extract, increasing the dose of the ioduret from two or three to twelve or twenty grains in the course of twenfour hours. Along with the cicatrization of the sores, the improvement of the general health was most remarkable; the appetite improved, the muscular strength increased, and the complexion acquired the florid hue of vigorous health. The salt, no doubt, is taken into the circulation, and acts on the blood itself, as well as on the capillary vessels in every part of the body.—*Ibid.*

* Med. Gazette, Sept. 4, 1840. † Ibid.

* Medical Gazette, September 11, 1840.

